# Spark Plasma Co-Sintering of Mechanically Milled Tool Steel and High Speed Steel Powders

**DOI:** 10.3390/ma9060482

**Published:** 2016-06-16

**Authors:** Massimo Pellizzari, Anna Fedrizzi, Mario Zadra

**Affiliations:** 1 Department of Industrial Engineering, University of Trento, via Sommarive 9, Trento 38123, Italy; 2 Iveco Defence Vehicles, Product Development & Engineering, via Volta 6, Bolzano 39100, Italy; anna.fedrizzi@cnhind.com; 3 K4Sint, via Dante 300–B.I.C., Pergine Valsugana 38057, Italy; mario.zadra@k4sint.com

**Keywords:** mechanical milling, hot work tool steel, high speed steel, spark plasma sintering

## Abstract

Hot work tool steel (AISI H13) and high speed steel (AISI M3:2) powders were successfully co-sintered to produce hybrid tool steels that have properties and microstructures that can be modulated for specific applications. To promote co-sintering, which is made difficult by the various densification kinetics of the two steels, the particle sizes and structures were refined by mechanical milling (MM). Near full density samples (>99.5%) showing very fine and homogeneous microstructure were obtained using spark plasma sintering (SPS). The density of the blends (20, 40, 60, 80 wt % H13) was in agreement with the linear rule of mixtures. Their hardness showed a positive deviation, which could be ascribed to the strengthening effect of the secondary particles altering the stress distribution during indentation. A toughening of the M3:2-rich blends could be explained in view of the crack deviation and crack arrest exerted by the H13 particles.

## 1. Introduction

Materials for tooling applications, such as tool steels, require a proper compromise between hardness and toughness to provide high wear resistance combined with adequate resistance to cracking. An increased wear resistance often comes at the expense of other properties, such as impact and fracture toughness. The possibility to produce hybrid materials with tailored properties in view of the specific application considered has been proposed as a valuable solution to overcome this problem [1,2,3].

Powder metallurgy (PM) is a technology that is suited to producing metal matrix composites (MMC). These materials consist of a tough metal matrix that is reinforced by a fine dispersion of hard particles (*i.e.*, carbides, nitrides, borides). To improve the resistance against grooving wear, hard particles must be larger than the abrasive medium [4], but as the hard particle size increases, the tensile and bending strengths of the MMC drastically decrease [5]. To minimize these negative effects, Berns et al. first proposed to reinforce the metal matrix with a harder steel instead of a ceramic material [5,6]. According to Berns’ considerations, the present authors also investigated the properties of a hybrid tool steel produced using spark plasma co-sintering (SPS) of a hot worked tool steel (HWTS) and a high speed steel (HSS) [7,8]. SPS is an electric field assisted technology in which a uniaxial pressure combined with a pulsed direct current are applied to produce fully dense materials in a shorter time and at a lower temperature than hot isostatic pressing [9,10,11].

Previous works showed that it is possible to modulate the hybrid steel properties by changing the composition of the blend [7,8]. However, the co-sintering behaviour of the two steels highlighted a detrimental interaction of the two components and hinders densification [8]. Specifically, the HWTS sinters at a slightly lower temperature than the HSS. The densification of the HSS component is, thus, hindered by the already sintered HWTS skeleton, resulting in the formation of large pores and a considerable decrease in the hardness and toughness. In this respect, a beneficial effect has been demonstrated through the use of small diameter particles, which minimize the interaction between the two components, and the blends achieve nearly full density and good properties [8,12,13].

Mechanical milling (MM) can be successfully used to reduce both the particle size and crystallite size [14,15,16,17,18,19]. These refinements enhance the sintering process, allowing the production of highly dense materials with better mechanical properties [18,19]. MM can be performed using different technologies [14,15,16]. In a planetary ball mill, the refinement results from the continuous impacts occurring between the powder particles and balls in the vial. During this high-energy process, the particles are repeatedly flattened, cold welded and fragmented [15]. All of these phenomena are responsible for the morphological and microstructural evolution of the powder [14,15,16,17], which can be summarized as follows. In the early stage, the soft and ductile metal particles are easily cold welded by the ball impacts and form large aggregates. This process increases the particle size and considerably changes the particle morphology, which becomes more flat and elongated than in the as-atomized state [15,17]. As the milling process proceeds, the powder particles are continuously strain hardened, becoming progressively less ductile. As a result, their fragmentation by brittle fracture is observed. In this stage, fragmentation prevails over cold welding so that the particle size begins to decrease and the powder shape becomes round again [15,16]. When the particle size becomes too small, the powder particles tend to aggregate again. The system then reaches an equilibrium state in which the agglomerative force and the fragmentation force are balanced. At this optimum stage, the particle size distribution is quite narrow and the mean particle size remains constant at the minimum value [15,16].

In this work, HWTS and HSS powders were mechanically milled to refine their particle size and microstructure. These MM powders were then blended to produce fully dense hybrid steels with different compositions. The blends and two base steels were consolidated using SPS to preserve the fine microstructure obtained during milling [18]. The density, hardness, toughness and microstructure were investigated and compared to those of unmilled blends [8].

## 2. Materials and Methods

Two commercial gas atomized powders, corresponding to standard grades AISI H13 and AISI M3:2 were used as HWTS and HSS, respectively. Their chemical composition is listed in Table 1.

The starting powders were spherical with 94 wt % of the particles and a diameter less than 350 µm. In both cases, the typical dendritic microstructure produced by rapid solidification could be observed [20].

**Figure 1:**
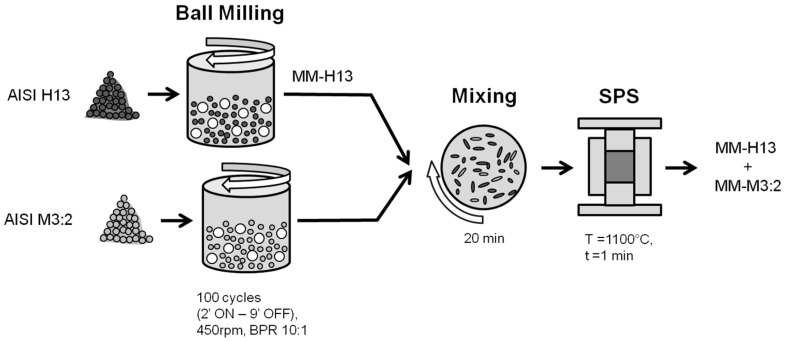
Schematic of the processing route to produce the hybrid tool steel by mechanical milling and SPS.

The route to produce the hybrid tool steel using MM and SPS is schematically represented in Figure 1. The MM was conducted in a Fritsch Pulverisette 6 planetary mono mill at 450 rpm under vacuum. Spheres of 100Cr6 (63HRC) with 10-mm diameters were used, and the ball-to-powder ratio was set to 10:1. To avoid overheating, cycles of 2 min on and 9 min off were used for a total milling time of 1000 min (500 cycles). These parameters were demonstrated to produce an optimum particle size and grain refinement in H13 [20]. The cumulative particle size distribution of the powders was measured using a “Partica LA-950^®^” (Horiba LTD, Kyoto, Japan) Laser Diffraction/Scattering Particle Size Distribution Analyzer. X-ray diffraction, using both Cu-kα and Mo-kα radiations, was used to identify the phase constitution of base powders and sintered materials.

Conversely, a similar systematic study of the milling conditions for M3:2 was not carried out and this grade was milled using the same parameters for H13. Two different milling runs were carried out for H13 and M3:2. Due to the lack of a suited protection system the powders pick up oxygen and nitrogen when opening the mill vial. The content of these two elements was measured using a LECO TC 400 Analyzer (LECO Corporation, St. Joseph, MI, USA).

Six samples containing different fractions of the two base materials were sintered (Table 2). The blended powders were mixed in a Turbola Mixer for 20 min.

Samples were finally consolidated in a DR. SINTER^®^ SPS1050 apparatus (Sumitomo Coal & Mining Co. Ltd., now SPS Syntex Inc., Tokyo, Japan). Disks with diameters of 30 mm and a 5-mm height were produced in graphite dies. SPS was carried out at 1100 °C with 1 min of isothermal holding at this temperature and final free cooling. The heating rate was 50 °C/min, and a compressive load of 42 kN, which corresponds to a pressure of 60 MPa, was applied once the temperature reached 600 °C. These sintering conditions were selected according to a previous study on the SPS of the as-atomized AISI H13 and AISI M3:2 powders [12]. The holding time was reduced to 1 min only to limit grain growth.

The density was measured using Archimedes’ principle according to ASTM B962-08 [21]. The relative density was calculated on the basis of the absolute density of the two MM materials measured using a pycnometer (ρMM-H13 = 7.71 g/cm^3^, ρMM-M3:2 = 7.97 g/cm^3^). The absolute densities of the four blends were calculated according to the linear rule of mixtures. After standard metallographic preparation and chemical etching, the microstructure of the milled powders and that of sintered materials was observed using scanning electron microscopy (ESEM, Philips model XL30, Philips, Eindhoven, The Netherlands).

All samples were vacuum heat treated by austenitizing at 1050 °C for 15 min and using 5 bar-N_2_ gas quenching and double tempering at 625 °C for 2 h each. Hardness was measured using a HV10 scale according to ASTM E92-82 [22]. The apparent fracture toughness, Ka, was determined using a procedure proposed for small fracture toughness specimens [23]. Notch the depth (a) with root radii (ρ) of 50 μm was electro-discharge machined in 6 × 3 × 30 mm^3^ (W × B × L) specimens. The ratio of the notch depth to the specimen width (*a*/*W*) was set at 0.5. Static fracture toughness testing was performed using a 10-ton capacity universal tester. The specimens were loaded in three-point bending at a crosshead speed of 0.5 mm/min according to ASTM E399 [24]. The properties of the current samples were compared with those of samples produced using unmilled powders [8].

## 3. Results

### 3.1. Mechanical Milling

**Figure 2:**
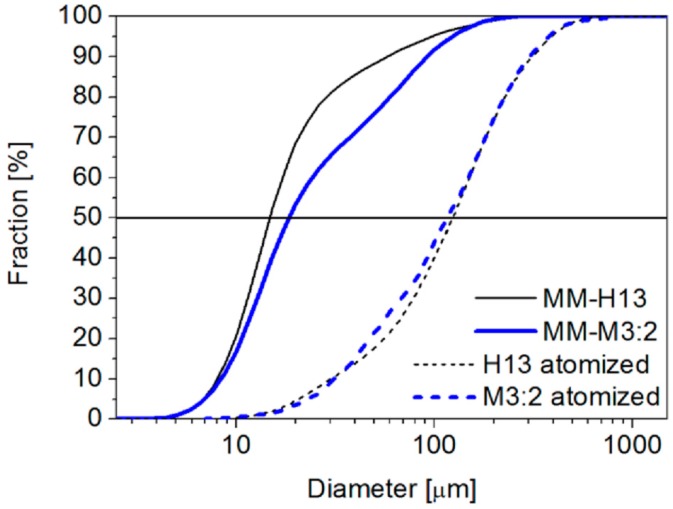
Particle size distribution of base as-atomized and mechanically milled powders.

A strong particle size refinement is observed in both steels as a result of the MM. The particle size distribution (Figure 2) demonstrates a decrease in the mean size from more than 100 µm (115 µm for H13, 123 mm for M3:2) to less than 20 µm (14.6 for H13, 18.3 for M3:2).

**Figure 3:**
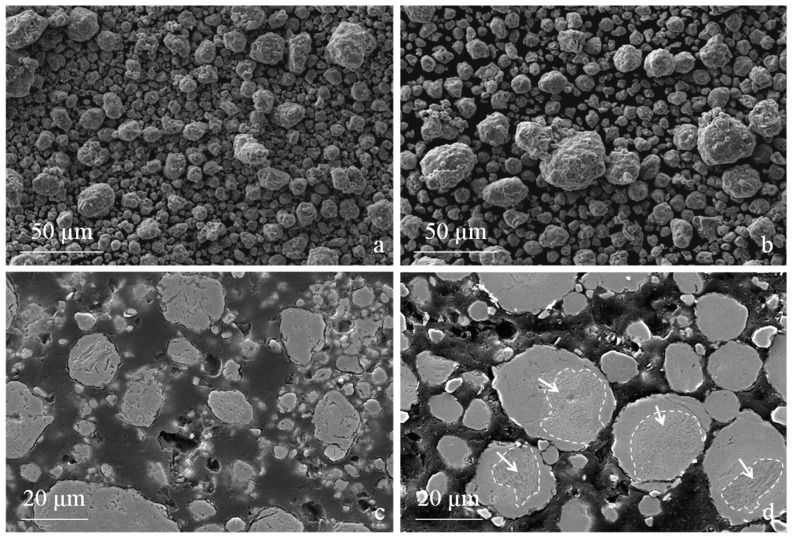
Microstructure (SEM) of the powders (**a**) AISI H13 and (**b**) AISI M3:2 milled for 500 cycles. Metallographic cross section of the same powders (**c**) AISI H13 and (**d**) AISI M3:2 at a greater magnification.

After only 100 cycles, the two powders showed a round morphology. This morphology did not change during the later stages of the process, as demonstrated by the powders milled for 500 cycles (Figure 3a,b).

A metallographic cross section highlights that some porosity was found inside the particles due to the repeated cold welding and fragmentation phenomena occurring during MM [14,15,16]. The same effects are also responsible for the disruption of the original inner solidification structure occurring by the stretching and deformation of the dendrites, leading to the formation of a lamellar microstructure [16,17]. As the milling time increased, the lamellae became closer and closer until the microstructure appeared to be fully homogenized. At the end of milling, no more traces of lamellar microstructure could be seen in the AISI H13 (Figure 3c). Conversely, the lower deformation of the MM-M3:2 particles still shows traces of a dendritic structure (see marked regions in Figure 3d), confirming that the MM conditions are far from the optimum and that a greater milling time must be considered to obtain better homogeneity.

**Figure 4:**
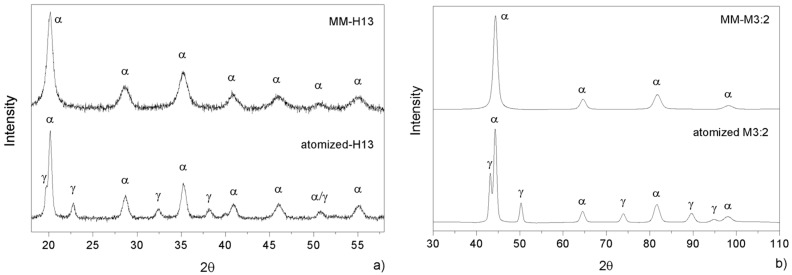
X-ray diffraction patterns for as-atomized and MM (**a**) AISI H13 and (**b**) AISI M3:2 powders.

Furthermore, MM was demonstrated to produce a strong structural refinement. A previous study showed that after 500 cycles, the crystallite size, which was measured by X-ray diffraction analysis, decreased from 74 nm to 12 nm in MM-H13 and that, according to the high dislocation density introduced during strain hardening, the hardness increased form 830 HV to 1380 HV [20]. Similarly, the crystallite size of M3:2 decreases from 50 nm to 14 nm in MM-M3:2. Moreover, in both steels, MM promotes the full strain induced transformation of retained austenite (Figure 4). Therefore, MM brings the material to a considerably greater free energy level compared with the original as-atomized state, *i.e.*, more distant from equilibrium, which is a very good starting condition for the faster sintering kinetics of difficult-to-sinter materials, such as those investigated here.

### 3.2. Spark Plasma Sintering

#### 3.2.1. Densification

**Figure 5:**
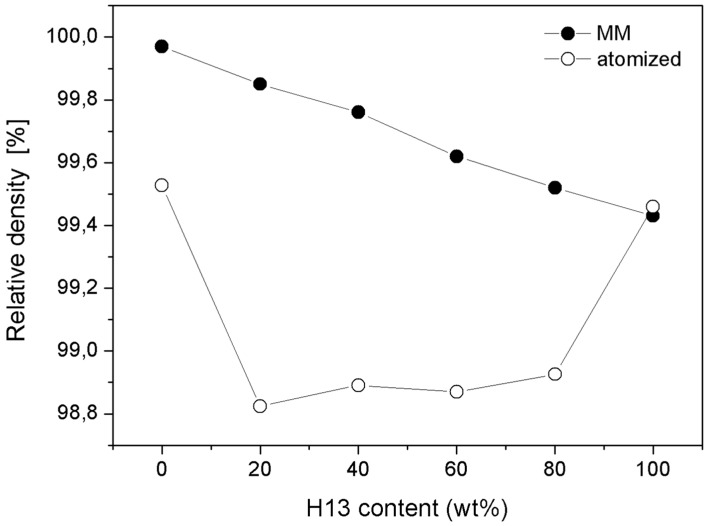
Density and relative density of the sintered specimens as a function of AISI H13 content.

The absolute density of the MM samples linearly decreased as the weight fraction of AISI H13 (*i.e.*, the component with the lower density) increased (Figure 5), which is in good agreement with the linear rule of mixtures. The relative density was calculated as the ratio to the density of the milled powders measured using a pycnometer. Because the milled powders have some internal porosity, especially milled AISI M3:2, these measures are thought to be lower than the theoretical density of the two MM steels. Therefore, the relative density values of the MM materials can be slightly greater than the real values, particularly for specimens with a greater amount of HSS, *i.e.*, MM-M3:2, MM-20H13 and MM-40H13. In any case, the present data confirm that all of the MM samples achieve near full density.

In the case of specimens fabricated using as-atomized powders, the relative density of all blends was less than the density of the two base steels, and all of these blends did not achieve the theoretical density predicted by the linear rule of mixtures. These specimens presented a large amount of porosity, which could be attributed to the different sintering kinetics of the two steels [8]. The sintering process of the as-atomized AISI H13 began at a lower temperature than in AISI M3:2, so the subsequent densification of the HSS was hindered by the presence of a rigid AISI H13 skeleton [8]. The present results confirm that this interaction is significantly minimized after reducing the particle size by MM. Indeed, small AISI H13 particles are less detrimental for achieving a high density because they exert a lower constraint on the AISI M3:2 sintering.

**Figure 6:**
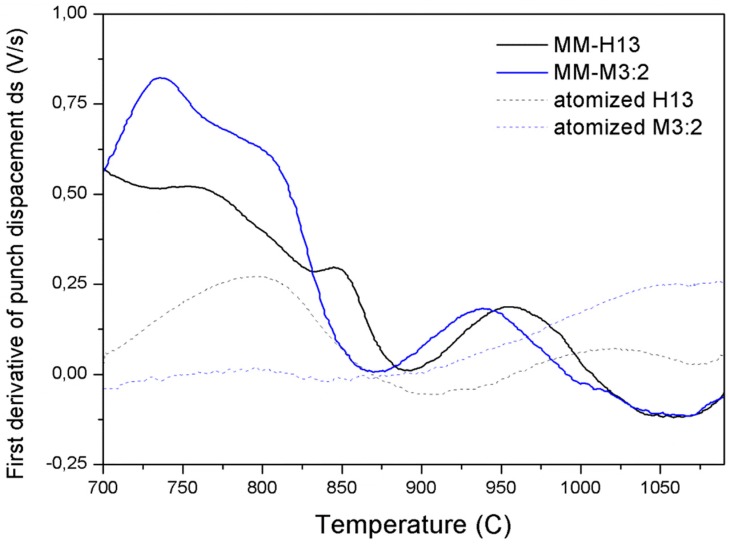
The lower punch displacement during the SPS of MM and the as-atomized base steels.

A confirmation can be found in the graph displaying the first derivative of the punch displacement during SPS as a function of temperature (Figure 6).

For a more detailed explanation of the form of this curve, the reader should see the authors’ previous papers [8,12,13]. For the purpose of the present discussion, it should be noted that this curve can be representative of the densification rate. When comparing the curves of the MM and as-atomized steels, the MM steel curves are observed to be shifted to a lower temperature, meaning that densification is activated by the milling process. The densification rate of the MM steels is very high, even at a low temperature (700 °C), where the rate of the atomized samples is practically zero. Furthermore, whereas the densification rate abruptly drops at 1050 °C, which is where the densification process of the MM steels is practically concluded, the same does not occur for the atomized steels. Finally, the curves of the MM steels are much closer, which is synonymous with having similar densification kinetics.

#### 3.2.2. Microstructure

**Figure 7:**
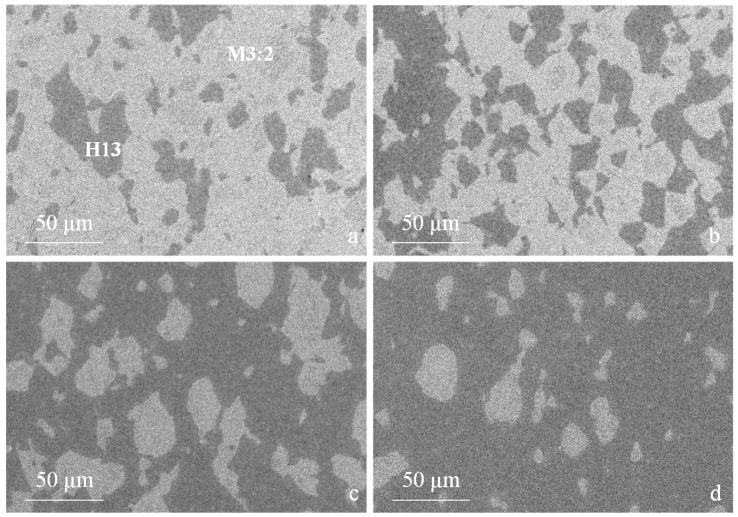
Microstructure of hybrid tool steels (SEM-BSE): MM-20H13 (**a**); MM-40H13 (**b**); MM-60H13 (**c**) and MM-80H13 (**d**). Light and dark regions correspond to M3:2 and H13, respectively.

After SPS the particles of the two steels are very well dispersed and the microstructures of the blends are quite homogeneous (Figure 7). The reduction in the particle size, especially of the largest particles, results in a more uniform microstructure than that produced using unmilled powders. Furthermore, in agreement with the density data, the MM-blends do not show any appreciable porosity which is instead very evident in the as atomized blends (Figure 5) [8].

**Figure 8:**
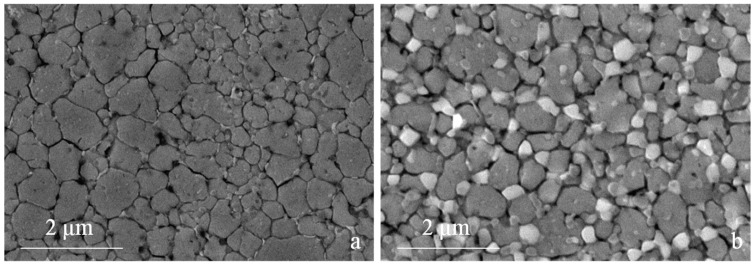
Microstructure of the two base MM steels (**a**) H13; (**b**) M3:2.

**Figure 9:**
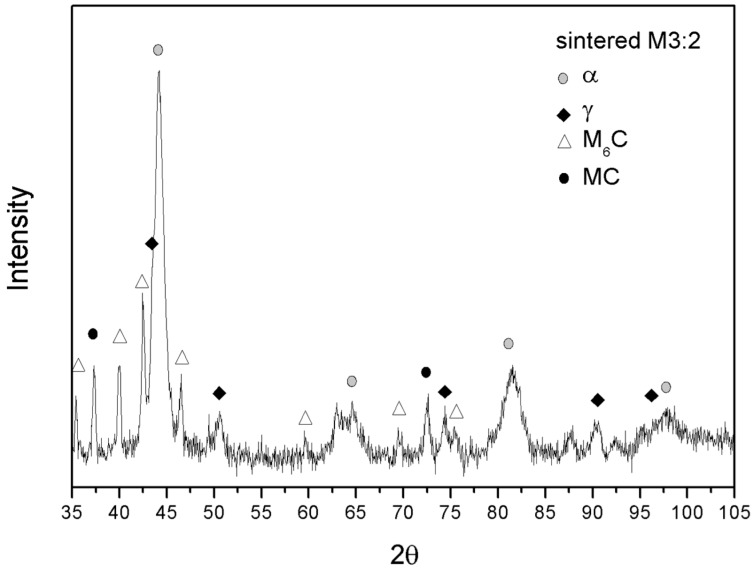
X-ray diffraction pattern for sintered M3:2.

**Figure 10:**
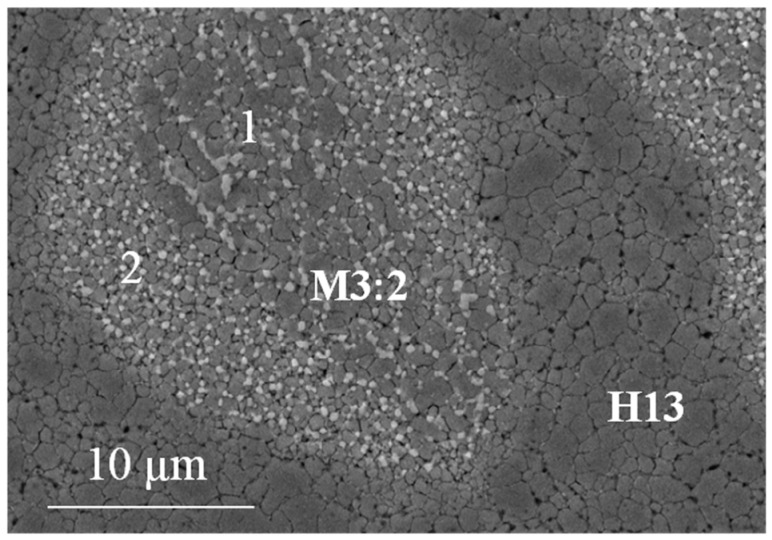
Microstructure of the hybrid tool steel showing the H13 and M3:2 regions. Please note the different grain sizes and carbide distributions in the outer (2) and inner (1) M3:2 particle regions due to the different extent of plastic deformation after milling (see also Figure 3d).

A closer look at the microstructure of the two base steels shows a much finer grain size compared with as-atomized samples [20]. The grain size indicates that recrystallization occurred during sintering but also that that grain growth could be limited to obtain an average grain size of 0.94 µm and 0.75 µm for H13 (Figure 8a) and M3:2 (Figure 8b), respectively. The present result confirms the suitability of SPS as an evaluable method for the consolidation of nanostructured powders. A quite impressive result is the very fine and homogeneous dispersion of MC (grey particles) and M6C (white particles) carbides in MM-M3:2, which are very effective in ensuring a very small and uniform grain size after sintering. Comparing the XRD patterns in Figure 4b and Figure 9 it can be inferred that these carbides precipitate during sintering. The positive influence of MM, in this respect, can be inferred from the microstructure of the larger HSS particles, which showed a less deformed inner part compared with the smaller particles. After sintering, the microstructure in the core region (1 in Figure 10) demonstrates a less intense carbide precipitation than in the outer shell (2 in Figure 10), resulting in a coarser grain size.

In co-sintered steels, this is reflected in a further interesting result, which is the refinement of the grain size of the H13 neighbouring the HSS particle, *i.e.*, showing the smallest grain size. In other words, the grain growth in H13 is constrained by the grain boundaries of M3:2.

#### 3.2.3. Hardness

**Figure 11:**
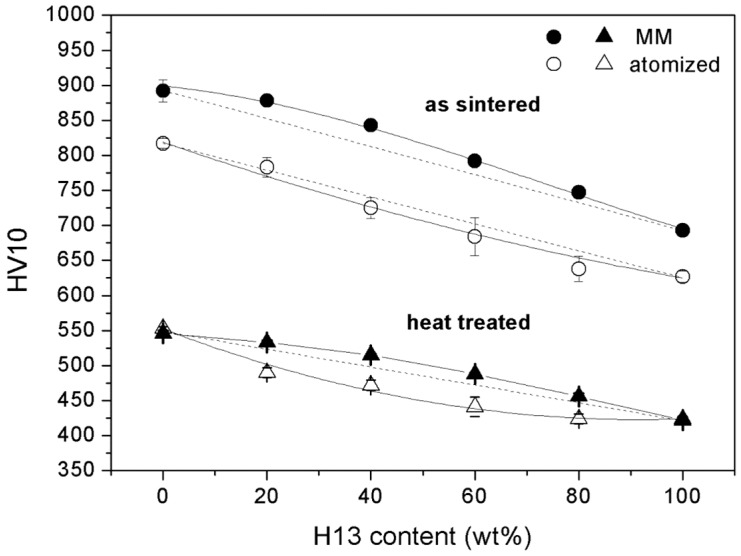
Hardness of the as-sintered and heat-treated hybrid tool steels.

The hardness of the MM samples was measured in the as-sintered and the heat-treated state (Figure 11). The high values in the as-sintered state are representative of the primary martensite microstructure forming during the post-SPS cooling stage. As discussed previously, the sintered samples still show the effects of MM, which are reflected in a greater hardness than the as-atomized ones. The temperature-time combination used for sintering plausibly preserves part of the straining effect previously induced by MM so that the hardness of the as-sintered samples (892 HV for M3:2 and 693 HV for H13) is approximately 75 HV greater than that of as-atomized samples (817 HV for M3:2 and 62 7 HV for H13). Further investigation is needed to distinguish any possible influence of the finer grain size from that of dislocations.

The hardness becomes considerably less after quenching and tempering at 600 °C to obtain the required balance between the hardness and toughness. Tempering above the secondary hardness peak was shown to substantially hide the structural modifications induced by the MM so that the atomized and MM samples of the two base steels show the same hardness [25].

As expected, a lower hardness was observed in AISI H13 due to the lower content of carbon and alloying elements (Table 1), which results in the formation of a softer martensite and a lower amount of carbides. The four MM blends achieve greater hardness values than those predicted by the linear rule of mixtures (dashed line in Figure 11). Previous investigations demonstrated that the dispersion of hard particles in the metal matrix increases the flow resistance and improves the hardness [26]. Furthermore, the investigation of the correlation between the hardness and the tensile strength highlighted that the slight improvement of the tensile strength resulting from the addition of hard particles may correspond to a comparatively greater increase in the hardness [27]. The greater work hardening of the MMCs could be traced back to the local compression of the metallic matrix and the greater concentration of hard particles in the loaded area [27]. The dispersion of hard particles also changes the stress distribution during loading so that stresses greater than the yield stress of the matrix are developed from the initial stage of indentation [26]. Further loading continuously increases the stress and strain in the matrix, causing further work hardening. Consequently, the MMCs show a greater work hardening rate than the metal matrix. For the blends present, the dispersion of the particles of a second constituent cause a similar modification of the stress field, resulting in increased work hardening of the matrix. Conversely, all of the blends produced by the as-atomized powders show a negative deviation from the rule of mixtures highlighting the negative influence of the poor densification [8].

#### 3.2.4. Fracture Toughness

**Figure 12:**
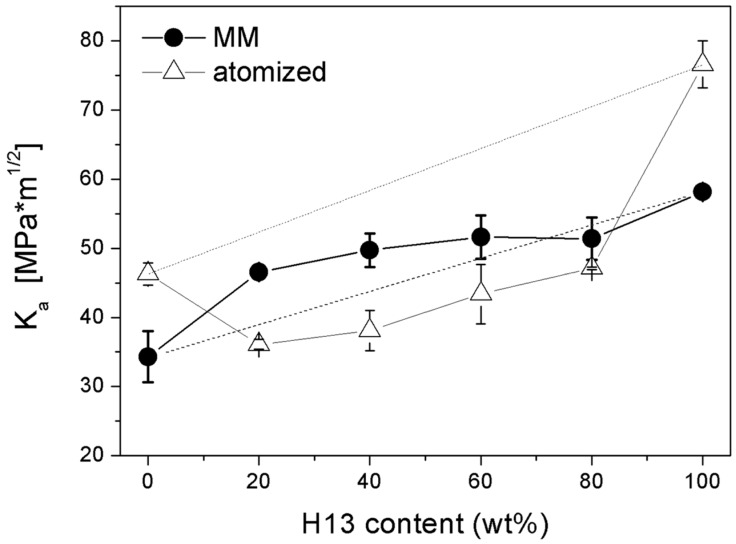
Apparent fracture toughness of tool steels from atomized and mechanically milled powders as a function of the AISI H13 content.

Figure 12 shows the apparent toughness of the MM samples. MM-M3:2 shows less toughness than MM-H13 according to its microstructure and greater hardness. The four MM blends achieve apparent toughness values between those of the two base steels. The values of the two blends containing a greater fraction of AISI H13 (*i.e.*, MM-80H13 and MM-60H13) are close to those predicted by the linear rule of mixtures (dashed line in Figure 12). Although the authors are aware that the fracture toughness of composite materials cannot be simply predicted by the rule of mixtures, the value calculated in this way is reported to highlight the theoretical reference behaviour of a mechanical mix of the two powders.

**Figure 13:**
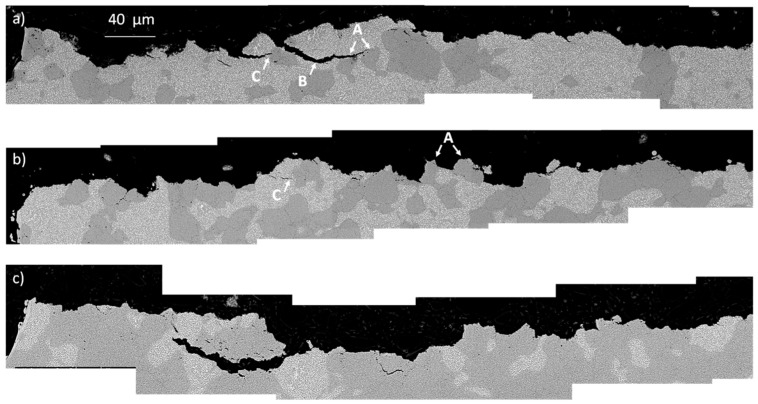
Cross sectional view of the fracture surfaces of (**a**) MM-20H13; (**b**) MM-40H13 and (**c**) MM-60% H13.

In MM-40H13 and MM-20H13, the addition of AISI H13 provides a positive deviation from the rule of mixtures, indicating a beneficial influence far beyond that expected by simple mechanical mixing. The reason for the relatively greater toughness of MM-40H13 and MM-20H13 has to be found in the mutual interaction between the two different powders. In the authors’ previous experience, AISI H13 produced by SPS generally shows interparticle fractures, resulting in a rough fracture surface [7,8]. Indeed, the same effect can play a toughening role when AISI H13 particles are placed in a less tough matrix. During fracture propagation, the H13 particles force the crack to deviate along their surface (details A in Figure 13a) instead of crossing the M3:2 particles (details B in Figure 13a).

This makes the crack path more winding and dissipates more energy, resulting in increased toughness. This explanation is in good agreement with the extent of the observed deviation, which decreases from 20% to 60% H13. By increasing the H13 content, the interparticle spacing progressively decreases, making the crack path less tortuous. In MM-80H13, the HWTS particles are practically interconnected and there is no benefit with respect to the fracture toughness that can be appreciated. Moreover, the H13 particles also act as a barrier against crack propagation (detail C in Figure 13c), suggesting a second possible toughening effect. Conversely, the M3:2 particles do not similarly obstruct the propagation of the crack in the H13-rich blends. Due to the lower toughness, intraparticle cracking is observed in M3:2 such that the crack in H13 proceeds almost straight across them without any toughening effect. Hence the toughness of blends with high H13 fraction decreases according to the linear rule of mixture.

Compared with the as-atomized materials (empty symbols in Figure 12), the apparent toughness of MM-M3:2 decreases from 46 Mpa·M^1/2^ to 34 Mpa·M^1/2^ and that of MM-H13 from 77 Mpa·M^1/2^ to 58 Mpa·M^1/2^. This can be explained in view of the oxygen pick-up shown by the powders after MM (Table 3).

Due to the lack of a suited insulation system, during post-milling operations (e.g., the delivery of powders to the SPS unit) the contact of highly reactive powders with the environment cannot be avoided, and the oxygen content increased almost one order of magnitude compared with the as-atomized powders (Table 1). The surface of the powders was covered by a thin oxide layer that impairs consolidation during sintering and reduces toughness [7,8,28]. This result suggests that if the oxygen content did not increase, the toughness of all of the MM materials would be 10–20 Mpa·M^1/2^ greater. However, despite of the negative effect of the greater oxygen content, all of the MM blends show greater toughness than the as-atomized blends, which exhibit a greater porosity. It can be concluded that the high porosity due to poor densification is more detrimental for toughness than a high oxygen content. In other words, as far as the present results are concerned, the benefits on densification by MM largely compensate for the detrimental effect of the greater oxygen content. Unquestionably, proper systems aimed at reducing oxidation could bring more benefits than those shown in this research.

## 4. Conclusions

Hybrid tool steels were successfully produced by mechanical milling and spark plasma sintering of AISI H13 and AISI M3:2 powders. MM markedly reduced the particle size, which minimized the negative influence of the different densification kinetics of the two steels. A high refinement and homogeneous microstructure could be observed for MM-H13, whilst the milling parameters still need to be improved for M3:2. Additionally, near full dense samples (relative density >99.5%) could be sintered for any blend composition.

The results obtained confirm that the properties of the hybrid steel can be modulated by changing the blend composition. The density, hardness and apparent toughness of the blends fall between the values measured for the two base steels according to the H13/M3:2 content. The density values are in good agreement with those predicted by the linear rule of mixtures. Indeed, the hardness of the blends is slightly greater because of the modified stress field distribution in the composite material and the greater local fraction of particles in the plastically deformed steel matrix. An interesting toughening effect by the H13 particles was observed in the MM-M3:2-rich blends. The beneficial effect could be ascribed to two different contributions, namely, the crack deviation and crack arrest exerted by well-dispersed (not interconnected) H13 particles.

The lack of suitable protection against oxidation for MM powders during post-milling operations caused a sharp increase in the oxygen content, resulting in a marked decrease in the toughness for the two base steels. Their toughness is much less than the samples produced using the as-atomized powders. In spite of this, the toughness of the MM-blends is greater than that of the as-atomized blends because the positive influence of a greater density largely compensates for the detrimental influence of the greater oxygen content.

## Figures and Tables

**Table 1:** Nominal composition of the powders (wt %).

Material	C	W	Mo	Cr	V	Mn	Si	O	N	Fe
AISI H13	0.41	-	1.60	5.10	1.10	0.35	0.90	0.0105	0.0383	Bal.
AISI M3:2	1.28	6.40	5.00	4.20	3.10	-	-	0.0163	0.0559	Bal.

**Table 2:** Composition and coding of the samples.

Sample Code	Composition (Weight Fraction)	
	AISI H13	AISI M3:2
MM-H13	1.0	0.0
MM-80H13	0.8	0.2
MM-60H13	0.6	0.4
MM-40H13	0.4	0.6
MM-20H13	0.2	0.8
MM-M3:2	0.0	1.0

**Table 3:** Oxygen and nitrogen content in the MM powders.

Material	O (wt %)	N (wt %)
MM-H13 powder	0.1702	0.0978
MM-M3:2 powder	0.1391	0.0950
